# Microglial Function across the Spectrum of Age and Gender

**DOI:** 10.3390/ijms18030561

**Published:** 2017-03-04

**Authors:** Jillian C. Nissen

**Affiliations:** Department of Pharmacological Sciences, Stony Brook University, NY 11794-8651, USA; jillian.nissen@stonybrook.edu; Tel.: +1-631-444-3055

**Keywords:** microglia, aging, gender, neurodegeneration, Alzheimer’s disease

## Abstract

Microglia constitute the resident immunocompetent cells of the central nervous system. Although much work has focused on their ability to mount an inflammatory response in reaction to pathology, recent studies have delved into their role in maintaining homeostasis in the healthy brain. It is important to note that the function of these cells is more complex than originally conceived, as there is increasing evidence that microglial responses can vary greatly among individuals. Here, this review will describe the changing behavior of microglia from development and birth through to the aged brain. Further, it is not only age that impacts the state of the neuroimmune milieu, as microglia have been shown to play a central role in the sexual differentiation of the brain. Finally, this review will discuss the implications this has for the differences in the incidence of neurodegenerative disorders between males and females, and between the young and old.

## 1. Introduction

Microglia are a distinct class of glial cells that are critical in mediating the innate and adaptive immune responses of the brain and spinal cord. They are the resident macrophage-like immune cells of the central nervous system (CNS), and are involved in processes essential for development and maintenance of neural homeostasis, managing injury, and subsequent repair [1]. Due to their morphology, microglial cells are described as being in a “resting” state under healthy conditions. This term is somewhat misleading, since in vivo imaging has revealed that microglia actively scavenge and monitor their CNS microenvironment with long, ramified processes for pathogens and damage [2]. These highly motile processes ensure that microglia can rapidly respond to any local disturbances; it is estimated that microglia can survey the entire brain parenchyma within one hour [3]. As microglia express major histocompatibility complex type II (MHC II) proteins, they represent immature antigen presenting cells when in the resting state [4]. Outside of their roles as immunocompetent cells, microglia are intimately involved in modulating synaptic plasticity, as they not only monitor synapses [5], but are responsible for the vital developmental process of synaptic pruning [6,7].

Microglial inflammatory responses to CNS pathologies have been extensively explored in the literature over the years. However, what has not been clearly elucidated is why there is a disparity in the prevalence of these neurodegenerative disorders among the young and the aged, and between men and women. Increasing evidence indicates that the reason for this difference may lie in variations of homeostatic microglial function between genders and during aging. Despite this, prior to the past decade, understanding of the role of microglia in the healthy brain was largely neglected. In this review, we discuss recent findings analyzing microglial behavior across the spectrum of age and gender, and the implications this has regarding the risk of developing neurodegenerative disease.

## 2. Microglial Changes throughout Aging

### 2.1. Microglia in the Developing Brain

Although microglia are physiologically and functionally very similar to mononuclear phagocytes, including macrophages, dendritic cells, and monocytes, they cannot be considered the exact CNS equivalent of these cells. Mononuclear phagocytes, which give rise to peripheral tissue-specific macrophages, are derived from the bone marrow [8]. As microglia and macrophages share a large number of common markers [9], it was originally considered that they came from the same source. It was later shown that microglia are derived from a primitive macrophage population produced by the yolk sac, and primarily colonize the brain prior to birth beginning at approximately Embryonic Day 8.5 in mice [10,11]. Infiltration of the CNS by microglia is dependent on the establishment of the brain vasculature, and relies on an active blood circulation [11]. It is due to this connection that it was posited that microglia were involved in angiogenesis, and it has indeed been shown that microglial depletion results in sparser distribution of vessels [12,13], and that soluble factors released from microglia are responsible for vessel branching [14]. These cells ultimately develop a heterogeneous morphological phenotype dependent on their location within the brain and their distance from the vasculature. In highly perfused areas, microglia adopt a more complex, ramified morphology [15], while conditions that diminish blood flow delay this process and result in more ameboid cells [16]. Under physiological conditions, microglia proliferate during embryogenesis and locally self-renew thereafter without significant contributions from the bone marrow [17,18].

The fact that microglia are present in the neonatal brain so early in development means that microglia mature alongside neurons, which supports the idea that microglia are crucial mediators of CNS wiring. Local circuit refinement through the selective removal of axon branches and synapses is known as pruning [19]; one of the most well-established facets of microglial functionality in the developing brain. Synapses form as a result of sensory input, and thus in the early postnatal days microglia can be seen to associate with synapses, with a subsequent loss of dendritic spines [20]. Microglia have been shown to actively phagocytose inappropriate synaptic connections, an activity that is critical in modeling the neural network [6,7,21]. Several mechanisms have been proposed to explain this function. One focused on the role of the classical complement pathway, as C1q localizes to developing synapses and can mark these synapses for phagocytosis by microglia in a C3-dependent manner [22]. C1q-null mice exhibited signs of epilepsy as well as enhanced synaptic connectivity, suggesting insufficient pruning by microglia [23]. Another potential mechanism could be through modulation of levels of synaptic adhesion molecules. Tissue plasminogen activator released by microglia has been implicated in reducing the levels of these adhesion molecules, which are essential for synaptic activity and stability [6]. The presence of microglia has also been associated with impaired axonal outgrowth, as their activation with lipopolysaccharide (LPS) resulted in diminished axonal extensions during development, while microglial depletion promoted the reverse [24]. Thus, it is evident that microglia play a critical role in the architecture of the developing brain.

### 2.2. Microglia in the Adult Brain

In the adult CNS, microglia comprise approximately 10%–15% of total cells, with slight variations across brain regions [25]. Once established, the microglial population is maintained by local self-renewal rather than through differentiation of infiltrating monocytes. Although both macrophages and microglia express colony stimulating factor 1 receptor (CSF1R), disruption of signaling through this receptor in mice leads to ~99% depletion of microglia while it merely diminishes macrophage numbers [18,26,27]. Microglial processes are highly dynamic in adult mice, and it is estimated that they could survey the entire brain parenchyma within one hour [28]. In the absence of pathologies, microglia tend to have long, ramified extensions, although these are found to be shorter and thicker in regions that lack a blood-brain barrier (BBB) [29,30].

It has long been discussed that upon encountering infection or injury, microglia undergo a process known as “activation”. This term is somewhat disingenuous as it implies that microglia are inactive in the healthy brain, and only become functional upon encountering perturbations to CNS homeostasis. Rather than considering microglia “resting,” it is more accurate to picture them as in a “surveying” state before adopting a reactive, “activated” phenotype. These changes can occur rapidly within minutes of encountering harmful stimuli, upon which they obtain a more amoeboid shape and proliferate [31,32]. This results in the upregulation of major histocompatibility complex (MHC) II, which allows for further recruitment of other inflammatory cells such as neutrophils, lymphocytes, and monocytes into the injury site [33]. Activated microglia can release tumor necrosis factor alpha (TNFα) and nitric oxide (NO), which promote neurodegeneration [34]. However, microglia also produce anti-inflammatory cytokines such as transforming growth factor beta (TGFβ) and interleukin 10 (IL-10), which are neuroprotective and support recovery following injury [35].

As is the case for any immune cell, an essential function of microglia is to be able to distinguish between targets to destroy and healthy cells to protect [25]. In the periphery, the presence of adaptive immune cells allows for a tailored response to a specific threat [36], but there is limited infiltration of helper T cells into the CNS. However, microglia have been shown to function as antigen presenting cells in multiple sclerosis [37]. The primary mechanism by which microglia respond to pathogens or injury is through pattern recognition receptors (PRRs), or soluble mediators released from degenerating neurons. Microglia express toll-like receptors (TLRs) 1–9 [38], and scavenger receptors to detect the presence of intracellular phosphatidylserine (PS) on the extracellular membrane of dying cells [39]. These different TLRs allow microglia to respond differently depending on the particular receptors expressed. For example, TLR4 interacts with bacterial cell wall components such as lipopolysaccharide, which promotes a strong inflammatory response by microglia [40]. TLR4, in concert with TLR2, can sense damage induced by ischemia and develop a response in microglia that results in larger infarct size [41,42]. TLR4 can also detect the release of heat shock protein (HSP) 60 from dying cells within the CNS, creating a positive feedback loop as inflammation among microglial cells promotes further neurodegeneration [43].

Aside from HSPs, dying neurons release a plethora of factors such as cytokines, chemokines, and nucleotides which recruit microglia to the injured area [44]. CCL2, also known as monocyte chemoattractant protein 1 (MCP1), is enriched in injured areas of the CNS. This protein is a chemokine that rapidly recruits microglia through binding to its cognate receptor CCR2 [45]. Disruption of CCL2–CCR2 signaling results in reduced hematoma volume in an animal model of intracerebral hemorrhage [46], and attenuates microglial migration and activation following excitotoxic injury [47]. One of the strongest signals between dying neurons and microglia is adenosine triphosphate (ATP), which can promote phagocytic activity and process extension in microglia [48]. ATP binds to purinergic receptors, of which microglia express seven variants [49]. The numbers of these receptors increase during aging, and are expressed in a sexually dimorphic manner dependent on subtype [50]. Activation of these receptors can be either deleterious or beneficial, as activation of the P2X_7_R variant on microglia promotes the release of inflammatory factors such as IL-1α/β [51], TNFα [52], and superoxide [53], but has also been shown to protect neurons against excitotoxicity resulting from glutamate exposure [54]. In the adult brain, microglia constantly survey for disruptions to homeostasis, and rapidly respond through induction of the inflammatory cascade, serving as the resident professional immune cell of the CNS.

### 2.3. Microglia in the Aged Brain

During aging, there is a wide variety of physiological changes that occur in the CNS. Brain weight decreases around 2%–3% each decade following the age of 50, and accelerates to a loss of about 10% in individuals over 80 compared with young adults [55]. In general, during the aging process, the immune system shifts to a chronic mild inflammatory state, with increased levels of pro-inflammatory TNFα and IL-1β in the CNS, and systemic increase of IL-6 [56,57], along with a concomitant decrease of the anti-inflammatory cytokine IL-10 [57]. Correlations of cytokine levels to cognitive ability have been inconclusive, but there is some evidence that very high levels of IL-6 could be related to a higher risk of cognitive decline [58]. As microglia are the specialized immune cells of the CNS, these impairments may reflect an alteration in their normal functions. CNS cells undergo a wide variety of genetic and functional changes during aging, as they show a gradual accumulation of DNA damage and oxidative stress over time [59]. This is particularly notable in microglia, as these cells are long-lived and not readily replaced by cells from the periphery [17]. As discussed above, since microglia are so intimately involved in maintaining the connectivity and health of the CNS in homeostatic conditions, any disruption of normal microglial function could have major impacts on the condition of the brain.

In young humans and animals, microglia in the healthy CNS are distributed evenly throughout the neural parenchyma, providing complete coverage of the entire brain and spinal cord. However, during aging, microglia have been seen to increase in both number and density in particular CNS compartments, including the visual and auditory cortices, hippocampus, and retina [60,61,62]. Why this accumulation occurs is poorly understood, although several hypotheses have been put forward. One suggests that it may be a compensatory mechanism for declining microglial function during aging [63], while others theorize that it may be due to failure of microglial numbers to completely revert back to basal levels after a lifetime of injuries, infections, and other damage [17]. However, these hypotheses still do not account for the preferential accumulation in specific site. They also undergo significant morphological changes, as aged microglia have smaller dendritic arbors, are more elongated, and are less circularly symmetric [62,64]. In humans, increased numbers of dystrophic microglia with processes that become fragmented and lose fine branches, are unusually tortuous, and are characterized by bulbous swellings appear during aging [56]. Using live cell imaging of mouse retinal microglia, it was shown that aged cells have slower process motility and reduced response to ATP. Notably, in response to a laser-induced injury aged microglia were both slower to migrate to the injury site and also resided there longer following recovery, supporting the above hypotheses for microglial accumulation [61].

**Figure 1:**
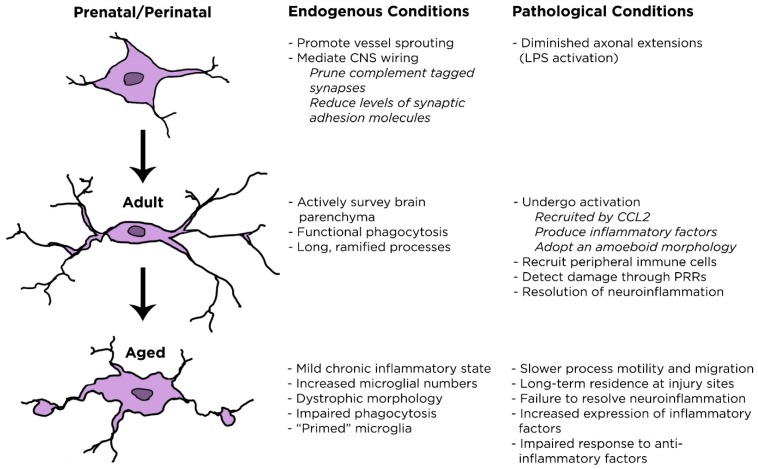
Age-related changes in morphology and function of microglial cells. CNS, central nervous system; LPS, lipopolysaccharide.

Microglia in aging brains upregulate a variety of “activation” markers, including MHC II, CD86, and PRRs including TLRs and nucleotide-binding oligomerization domain-like (NOD)-like receptors [65,66]. Levels of pro-inflammatory cytokines are increased as well, such as IL-1β, TNFα, and IL-6 [67,68]. Observations of these alterations has led to the idea that microglia become sensitized, or “primed” as aging progresses [69]. In fact, aged microglial responsiveness to peripheral signals and the local environment is exacerbated compared to young controls. When aged mice received an intraperitoneal injection of LPS at a dose that results in an acute mild sickness in young mice, their induced IL-1β production was significantly increased [70]. Aged mice also exhibit increased hippocampal microglial numbers following LPS injection compared to their young counterparts [71]. In parallel to the fact that aged microglia tend to linger in injury sites [61], aging rats injected with *Escherichia coli* have increased hippocampal IL-1β protein levels that remain high for longer than young rats [72]. Interestingly, aged mice expressed higher levels of IL-10 than young mice [64]. IL-10 is an anti-inflammatory cytokine that regulates the production of IL-1β, so it is possible that aged mice lose responsiveness to IL-10. The shift of microglia to a more sensitized, “primed” state could imply that these cells would respond much more rapidly and to a greater degree to a secondary stimulus. Thus, as the brain ages, microglia become more reactive, more inflammatory, and increasingly dystrophic, while showing resistance to anti-inflammatory signals. Microglial changes throughout aging are summarized in Figure 1.

## 3. Microglial Differences between Genders

### 3.1. Sexual Differentiation of the Brain

Sex determination in mammals is defined by the presence of the Y chromosome gene *Sry*, which is obligatory for proper development of the testes through coding of the testis determining factor. In the absence of this gene, regardless of how much else of the Y chromosome is present, the gonads instead develop into ovaries [73]. Following this process of sex determination, which is defined by the generation of sex-specific gonads, sex differentiation in other tissues occurs.

The fetal gonad develops early during gestation, and in primates androgen production is evident by the end of the first trimester [74]. Following entry into the brain, testosterone can be processed into either estradiol or dihydrotestosterone (DHT). Testosterone is essential in masculinizing the brain, as exposure of female guinea pigs to testosterone in utero permanently disrupted their ability to display female reproductive behaviors. However, there is a critical period for this modulation, as exposure to testosterone in adulthood does little to affect these behaviors [75]. Estradiol, which is a masculinizing hormone during development in rodents, could potentially be responsible for mediating some of the gross anatomical differences in male and female brains. This hormone is capable of promoting neurite outgrowth in organotypic explant cultures from the preoptic area, hypothalamus, and cerebral cortex [76]. The preoptic area (POA), a major brain region involved in male sexual behavior [77], exhibits a density of dendritic spine synapses in males that are twice as abundant as found in females [78]. This brain region is ultimately 5–7 times larger in males than in females at maturity [79]. Transgenic animals with XX chromosomes that developed testes through incorporation of the *Sry* gene on autosomes (XX-males) had similar neuroanatomical brain structures and social behaviors to wild-type XY males, and showed significant differences to wild-type XX females [80]. This result indicates that the presence of the gonads, and by proxy sex-associated hormones, is responsible for sexual differentiation of brain configuration. However, using the same mouse model it was found that XY-males were more aggressive than XX-males, and XX-females showed stronger parenting behaviors than XY-females (animals with *Sry* deleted from their Y chromosome) [81], demonstrating that hormone effects could not explain the entirety of neural sexual dimorphism.

Of note is that the X chromosome has the highest concentration of immune-related genes throughout the entire genome, which could potentially explain why certain autoimmune disorders are so prevalent in females [82]. Multiple sclerosis, a CNS autoimmune disorder which is diagnosed in women over men at a 3:1 ratio [83], has been investigated through an animal model known as experimental autoimmune encephalomyeltitis (EAE). This female prevalence has been supported by studies that show that gonadectomized XX-mice of both genders show greater deficits in EAE than gonadectomized XY-mice [84]. As microglia are the primary immune cell of the CNS, it leads to the question of whether these cells are sexually dimorphic in their function as well.

### 3.2. Gender Differences in Microglial Function

Microglia are found in an activated state in the early postnatal period, and return to a resting state by the third week following birth [21,85]. By Postnatal Day 4, males have greater numbers of amoeboid microglia in the hippocampus, amygdala, and cortex in comparison to females, which diminishes by adolescence [85]. This window corresponds to the “critical period” of hormonally-mediated sexual differentiation of the brain, as an androgen surge results in increased levels of estradiol in the brain that results in masculinization in males [75]. A critical mediator of this masculinization process is prostaglandin E2 (PGE2), which is downstream of estradiol signaling in the POA [86]. PGE2 promotes the formation and stabilization of dendritic synapses, which results in the larger POA seen in males [87]. Microglia in the male POA produce more PGE2 than those in females, and if females are treated with PGE2 this can masculinize their brain [88]. Further, microglial activation is necessary for development of the male features of the POA, as treatment with minocycline, a microglial inhibitor, disrupts the ability of estradiol to promote a male phenotype in dendritic spine numbers [88]. Beyond this correlation, increasing evidence has shown that it is not just microglial numbers that are not comparable between males and females, but that their activity and signaling differ as well.

Primary microglia extracted from the whole brain of female mice at Postnatal Day 3 have higher expression levels of the inflammatory cytokines TNFα, IL-1β, and IL-6 compared to males, but this disparity was resolved by Postnatal Day 21 [89]. Again, this timing correlates to the critical period of hormonal expression for brain sex differentiation. There are also variation in purinergic receptors between male and female microglia, as in the early postnatal period males express lower levels of P2X_4_ and P2Y_4_ receptors [50]. Purinergic receptors are generally associated with microglial activation, and loss of this signaling can disrupt the transition from a quiescent to an inflammatory phenotype [90]. These differences are also recapitulated in injury models, as cortical lesion sites are predominated by either resting or anti-inflammatory microglia in males, while the opposite is the case for females [91]. Taken together, it is suggested by these data that the neuroimmune milieu in females is more inflammatory than males in both the healthy and injured states.

### 3.3. Hormonal Regulation of Neuroimmune Function

Many of these variations between male and female microglia could potentially be explained by differential signaling by steroid hormones, for which microglia express receptors. Several studies have demonstrated the expression of one or both estrogen receptor (ER) subtypes, ERα and ERβ, on microglial cells [92,93]. ERβ, which has had conflicting reports regarding its expression on microglial cells [93,94], was found to be highly expressed on the BV-2 microglial cell line, more so than on the N9 microglial cell line [92]. The original source of N9 cells was male mice, and BV-2 cells from females, so it is conceivable that this variant of the estrogen receptor may be enriched in females [95]. At three and seven weeks of age, microglia isolated from male cortices expressed higher levels of ERα than females [96].

**Figure 2:**
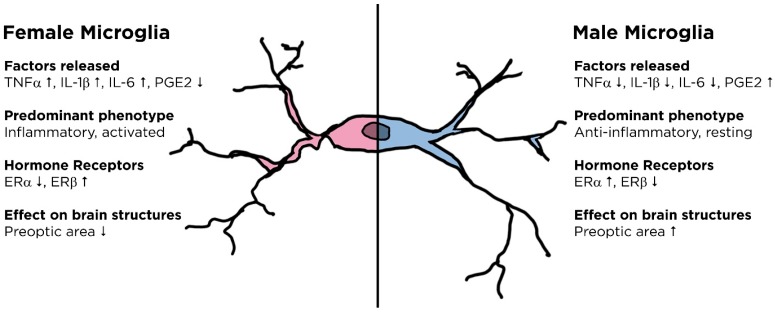
Gender-related disparities in microglial function. All comparisons are relative to microglia of the opposite gender. TNFα, tumor necrosis factor alpha; PGE2, prostaglandin E2; ER, estrogen receptor.

Hormones greatly influence microglial function, as treatment of rat microglial cells with 17β-estradiol, the major female sex steroid following development, results in an inhibition of phagocytosis and diminished levels of reactive oxygen species (ROS) production, inducible nitric oxide synthase (iNOS) expression, and PGE2 release [94]. Interestingly, estradiol has a sexually dimorphic effect on IL-1β production. Culturing male microglia in the presence of estradiol has an anti-inflammatory effect, as this significantly reduces their IL-1β production. Surprisingly, estradiol produces an inflammatory response in female microglia instead. This was further supported through the observation that female rats supplemented with estradiol in vivo produced a potentiated IL-1β response among microglial cells compared to males. [97]. It is not yet understood if the prevalence of ERα in males and ERβ in females contributes to this disparate response. These data correlate to observations discussed above, as it demonstrates that hormonal signaling further promotes the inflammatory response in female microglia while exerting an anti-inflammatory effect on those in males. Sexual dimorphism of microglial function is summarized in Figure 2.

## 4. Implications in Neurodegenerative Diseases

Neurodegenerative diseases, which include Alzheimer’s disease (AD), result from a complex interplay of factors that ultimately involve the interaction of the immune system and neurons. The brains of AD patients are characterized by the presence of aggregated Aβ plaques and neurofibrillary tangles, which contribute to neuronal loss that manifests in senility and physical disability [98]. Microglia are capable of clearing these Aβ aggregates through phagocytosis, although their capacity to do so is heavily determined by age and gender.

Age is a major risk factor for AD, with the likelihood of developing this disease increasing over time [99]. Further, AD is more prevalent among women than men [100]. Pro-inflammatory conditions promote the development and progression of AD [101,102], and treatment with non-steroidal anti-inflammatory drugs (NSAIDs) not only reduces the risk of developing AD, but also delays its onset [103]. This is possibly due to the fact that the phagocytic capacity of microglia is reduced in response to inflammatory cytokines [104], indicating that microglia committed to an inflammatory phenotype may be deleterious. Indeed, treatment of animals with minocycline, which suppresses microglial activation, significantly improves symptoms in an animal model of AD [105]. There is an age-associated impairment of phagocytosis in microglia [79], which results in further plaque accumulation. Microglia isolated from neonatal or young adult mice could effectively clear Aβ aggregates, but those isolated from six-month-old mice could not. However, older microglia more readily adhered to plaques than those isolated from young animals, so it is perhaps impairment in the actual engulfment process that causes this buildup [106]. On the other hand, evidence has shown that microglia become more phagocytic under inflammatory conditions, as they must boost their capacity to clear cells that have undergone apoptosis [107]. This contradicts studies that observe impaired phagocytosis in the presence of inflammatory cytokines. It may partially be explained by the fact that under endogenous conditions microglia can phagocytose apoptotic cells within 1.5 h, which increases to 6.3 h following seizure induction [30,107]. Thus, while microglia highly populate injury sites and can be observed to phagocytose cell debris, this process is in fact slower than during homeostasis.

In a general sense, based on the data presented above it can be thought that female microglia are more inflammatory than male, and aged microglia are more inflammatory than those from young animals. Thus, when assessing age and gender as two parameters related to a spectrum of immune activation states, young males conceivably would have the least inflammatory immune environment, with older women having a higher propensity towards microglial activation and production of inflammatory factors. As inflammation promotes a more rapid and severe AD course, it is logical that aged women would be the most likely to develop this disease, which correlates with epidemiological data from the clinic.

## 5. Conclusions

Taken together, it is clear that there is not a “one size fits all” for microglial function. A person’s age and gender can greatly impact their neuroimmune activity. As advanced age and female gender are both associated with increased chronic inflammation, these individuals are more likely to be susceptible to neurodegenerative disorders in the CNS such as AD. Further, this predominance in women is also recapitulated in several other CNS pathologies, including autoimmunity [108] and stroke [109]. Current disease therapeutics do not account for person-to-person variation in immune phenotype and function, which may account for why women tend to exhibit more rapid cognitive decline following diagnosis of AD [110]. Understanding how these parameters impact disease progression and recovery would bring about a whole new paradigm of age- and gender-specific therapies that could be tailored to individual patients.

## Abbreviations

AD	Alzheimer’s disease
ATP	adenosine triphosphate
BBB	Blood-brain barrier
CNS	Central nervous system
CSF1R	Colony stimulating factor 1 receptor
DHT	Dihydrotestosterone
ER	Estrogen receptor
HSP	Heat shock protein
IL	Interleukin
iNOS	Inducible nitric oxide synthase
MCP1	Monocyte chemoattractant protein 1
MHC	Major histocompatibility complex
NO	Nitric oxide
NOD	Nucleotide-binding oligomerization domain-like
PGE2	Prostaglandin E2
POA	Preoptic area
PRR	Pattern recognition receptor
ROS	Reactive oxygen species
TGFβ	Transforming growth factor beta
TNFα	Tumor necrosis factor alpha
